# Autoimmune disease-associated pathobionts: mechanisms and therapeutic potential of phage-based approaches

**DOI:** 10.3389/fimmu.2026.1884094

**Published:** 2026-09-01

**Authors:** Kentaro Kuzuya, Yuichi Maeda, Kenji Funakoshi, Yumiko Mizuno, Kosuke Fujimoto

**Affiliations:** 1 Department of Microbial Regulation, Research Institute for Microbial Diseases, The University of Osaka, Osaka, Japan; 2 Department of Respiratory Medicine and Clinical Immunology, Graduate School of Medicine, The University of Osaka, Osaka, Japan; 3 Department of Immunology and Genomics, Osaka Metropolitan University Graduate School of Medicine, Osaka, Japan; 4 Division of Metagenome Medicine, Human Genome Center, The Institute of Medical Science, The University of Tokyo, Tokyo, Japan

**Keywords:** autoimmune diseases, bacteriophage, endolysin, microbiota, pathobiont

## Abstract

The gut microbiota is a critical regulator of systemic immune homeostasis; accumulating evidence implicates specific commensal bacteria, termed “pathobionts,” in autoimmune disease pathogenesis. However, the definition of pathobionts remains context-dependent, as their effects are influenced by host genetics and host–microbe interactions. In this review, we summarize representative pathobionts supported by functional evidence in selected extraintestinal autoimmune diseases and discuss how these mechanisms may inform phage-based microbiome-targeted interventions. Mechanistically, pathobionts contribute to autoimmune disease through multiple pathways, including molecular mimicry, induction of intestinal T helper 17 and T follicular helper cell responses, disruption of regulatory T cell homeostasis, intestinal barrier dysfunction, and bacterial translocation from the gut to extraintestinal sites. These processes highlight the central role of gut-associated lymphoid tissue in initiating systemic autoimmunity, and targeting disease-associated microbes represents a promising therapeutic strategy. Whole-phage therapy, which enables highly specific bacterial elimination, has shown efficacy in preclinical immune-mediated disease models, but may be affected by variable *in vivo* replication, bacterial receptor-mediated resistance, anti-phage immune responses, and ecological effects on the resident microbiome. Phage-derived enzymes that lyse bacterial cell walls, such as endolysins, represent a complementary therapeutic modality that specifically targets bacterial peptidoglycan through cell wall-binding and catalytic domains. Collectively, these findings support the concept that pathobiont-targeted interventions, particularly phage-based strategies, may provide microbiome-directed, immunosuppression-sparing therapeutic approaches for selected patient subsets.

## 1 Introduction

Bacteriophages are viruses that specifically infect bacteria; they are the most prevalent biological entities on Earth, and are integral to the structuring of microbial communities, including those within the human body (1, 2). It is estimated that hundreds of trillions of bacteriophages inhabit the human body, surpassing both bacterial and human cells (3). These phages predominantly reside in areas of bacterial colonization, such as the intestine, skin, urogenital tract, and upper respiratory tract (4). The gut microbiome serves as the largest phage reservoir, containing approximately 10^8^–10¹^0^ phage particles per gram of human feces (5). Bacteriophages have been extensively studied since their discovery over a century ago. Recent next-generation advancements in metagenomic sequencing have facilitated a comprehensive characterization of phage diversity and ecological dynamics (6), elucidating their interactions with their bacterial hosts as well as their contributions to human health and disease.

Phage therapy, which selectively targets and eradicates specific bacterial pathogens, has yielded promising clinical outcomes in the treatment of various multidrug-resistant infections (7), including those caused by *Pseudomonas aeruginosa* (8) and *Mycobacterium abscessus* (9). Unlike broad-spectrum antibiotics, which potentially disrupt commensal microbiota, phage therapy offers highly specific antimicrobial activity, enabling targeted bacterial elimination while minimizing dysbiosis (10). Although certain bacterial species play crucial roles in non-infectious diseases, the application of phage therapy in this context remains unestablished. Extending phage-based interventions to chronic immune-mediated diseases will necessitate not only the identification of disease-relevant bacterial targets but also a deeper understanding of host immune recognition of phages, treatment durability, and the ecological consequences of selective microbial depletion.

Certain commensal bacterial species, often referred to as “pathobionts,” play a role in disease pathogenesis. The use of this term requires careful consideration; a recent review highlighted concerns about its frequent application to taxa identified through sequence-based associations with disease without establishing causality or elucidating mechanisms of virulence, which may lead to misinterpretation (11). Additionally, growing evidence indicates that bacterial strains labeled as pathobionts may have context-dependent effects, including beneficial roles, depending on host genetic susceptibility and interactions with the resident microbiota. *Enterococcus faecalis* can act as both colitogenic and protective, contingent on the composition of the gut microbial community (12). Similarly, *Helicobacter hepaticus* induces colitis in specific-pathogen-free IL-10-deficient mice but not in infected wild-type mice (13), and it produces a large polysaccharide that induces an anti-inflammatory gene program in macrophages (14). These findings emphasize the need for functional validation, indicating that studies confirming virulence are essential to define a microorganism as a true pathobiont (15).

**Figure 1:**
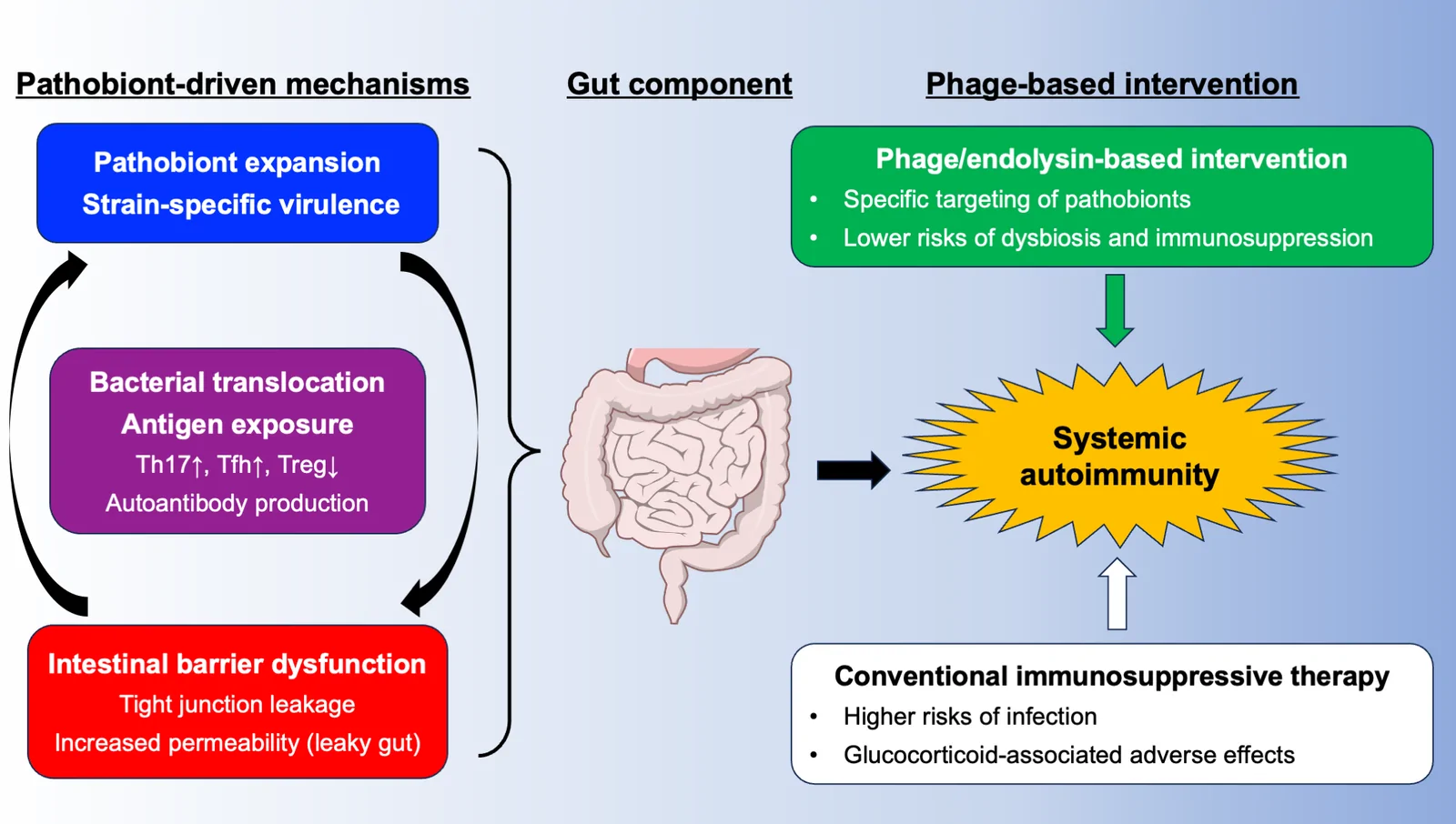
Conceptual framework of pathobiont-driven systemic autoimmunity and phage-based intervention. Pathobionts can contribute to the development of systemic autoimmunity by compromising the intestinal barrier, facilitating bacterial translocation, and initiating gut immune responses. Phage-based strategies, especially those employing phage-derived enzymes, offer a targeted method to address disease-associated microbes while preserving the balance of the commensal microbiota. Phage-derived enzymes may complement whole-phage therapy by enabling enzyme-based, pathobiont-directed bacterial depletion. The illustration was created using elements from NIAID NIH BioArt Source entry 212.

In this review, we do not aim to cover all autoimmune diseases in which microbiota alterations have been reported. Instead, we focus on selected extraintestinal autoimmune diseases in which candidate pathobionts are supported by functional or mechanistic evidence beyond sequencing-based associations. Gut microbiota-related mechanisms have also been reported in other autoimmune diseases, including multiple sclerosis (16), type 1 diabetes (17), and Sjögren’s syndrome (18); pathobionts in inflammatory bowel disease have been comprehensively reviewed elsewhere (19). We review representative experimentally validated pathobionts associated with systemic autoimmune diseases and use these examples to provide the basis for the mechanistic and therapeutic discussions in subsequent sections (**Supplementary Table 1**). This conceptual framework, in which gut pathobionts promote systemic autoimmunity through barrier dysfunction, bacterial translocation, and gut immune activation, and may be selectively targeted by phage-based approaches, is summarized in **Figure 1**.

## 2 Pathobionts in autoimmune diseases

### 2.1 Rheumatoid arthritis

Rheumatoid arthritis (RA) is a chronic systemic autoimmune disease characterized by persistent synovitis, followed by cartilage destruction and bone erosion, which can impair quality of life. The development of anti-citrullinated protein antibodies and rheumatoid factor can precede clinically apparent arthritis, suggesting that disease-related immune responses may be initiated outside the joint before synovitis becomes established (20). Mucosal sites, including the oral cavity, lung, and intestine, have been proposed as potential sites of early immune priming in RA, where microbial dysbiosis may promote the expansion of autoreactive immune responses (21). This mucosal-origin model provides the rationale for investigating gut pathobionts.

*Segatella copri*, previously known as *Prevotella copri*, is a well-characterized pathobiont linked to the intestinal initiation of arthritogenic T-cell responses in RA. Research on the human microbiome has consistently associated the prevalence of *S. copri* with RA, with an increased presence of *S. copri* seen in patients with RA in both Japan and the United States (22, 23). This implies that *S. copri* may contribute to disease onset in genetically predisposed individuals, rather than merely indicating dysbiosis. Gnotobiotic experiments with SKG mice have demonstrated that colonization with fecal microbiota from patients dominated by *S. copri* exacerbates arthritis compared with microbiota from healthy individuals, suggesting that the microbial community associated with RA has a functional capacity to promote disease (23). Further genomic analysis has revealed a conjugative transposon-associated region enriched in arthritogenic *S. copri* strains, indicating that strain-level genetic variations may enhance immunogenicity (24). Mechanistically, RA patient-derived *S. copri* induced higher interleukin 6 production by dendritic cells and promoted T helper (Th) 17 cell differentiation more strongly than healthy control-derived *S. copri* (24). Another study showed that outer membrane vesicles from *Palleniella intestinalis*, a member of the *Prevotellaceae* family, increased arthritis incidence by activating colonic CD11b^+^CD11c^+^ myeloid cells and promoting Th17 differentiation in an interleukin 6-dependent manner (25). Collectively, these findings suggest that RA-associated *Prevotellaceae* can promote mucosal innate immune activation and Th17-skewed autoimmune responses in a manner shaped by microbial community context and host susceptibility.

### 2.2 Systemic lupus erythematosus

Systemic lupus erythematosus (SLE) is a female-predominant systemic autoimmune disease characterized by loss of immune tolerance, autoantibody production against nuclear antigens, and immune-complex formation, which can result in multi-organ damage. Dysregulated innate and adaptive immune responses, particularly type I interferon signaling, autoreactive B-cell activation, T-cell dysfunction, and impaired clearance of nucleic acid-containing cellular material, are central to SLE pathogenesis (26). Increasing evidence suggests that the gut microbiome may contribute to SLE by promoting pathobiont expansion, microbial translocation, molecular mimicry, and immune cross-reactivity against microbial and self–antigens (27). These features provide a mechanistic rationale for examining translocating or immunostimulatory gut bacteria as potential contributors to SLE pathogenesis.

*Enterococcus gallinarum* has been identified as a pathobiont in SLE because it is capable of systemic translocation (28). *E. gallinarum* has been found in the mesenteric veins, mesenteric lymph nodes, and liver in lupus-prone (NZW × BXSB) F1 mice, with PCR-based detection in the liver tissues of patients with SLE (28). These findings shift the focus from compositional dysbiosis to functional attributes, such as epithelial barrier breaches and tissue infiltration. Supporting this perspective, conventional 16S rRNA sequencing of fecal samples has not necessarily indicated *E. gallinarum* enrichment (28).

Vaccination against *E. gallinarum* suppresses bacterial growth and ameliorates disease progression in lupus-prone mice, suggesting that this organism plays an active role in disease pathogenesis (28). *E. gallinarum* promotes Th17 polarization in human PBMCs and serum IgG3 antibodies against its RNA correlate with anti-human-RNA autoantibodies and disease activity in patients with SLE, implicating the pathobiont in both cellular and humoral autoreactivity (29). *E. gallinarum* exemplifies a broader principle in lupus microbiome research, demonstrating that pathogenic potential may be more closely related to translocation and immune activation capacity than abundance. *E. gallinarum* undergoes host-specific adaptive evolution within mammals, potentially enhancing its systemic dissemination and immune activation abilities (30). However, the mechanisms driving its translocation propensity and strong immunostimulatory effects remain incompletely understood.

*Ruminococcus gnavus* has also been identified as a pathobiont in patients with active lupus nephritis. Azzouz et al. reported an expansion of *R. gnavus* at the species level in patients with SLE and found that serum antibody responses to the organism correlate with severe (class III or IV) lupus nephritis (31). Transplantation of an SLE-derived *R. gnavus* strain into antibiotic-treated mice resulted in increased autoantibody production and intestinal permeability, with a female predominance (32). In a separate study, colonization with the distinct RG2 reference strain further promoted autoantibody production and T cell dysregulation in lupus-prone mice, supporting the notion that *R. gnavus* contributes to barrier dysfunction (32) and sustained immune imbalance (33).

An increased abundance of *Streptococcus intermedius* and *Streptococcus anginosus* has been reported in the gut microbiota of patients with SLE (34); metagenomic analysis revealed enrichment of *Streptococcus*-derived genes, including those involved in redox reactions, and microbiome–metabolome association analysis showed a positive correlation between acylcarnitine levels and *S. intermedius* abundance.

At least three complementary microbial mechanisms appear to be involved in lupus: translocation-driven immune activation exemplified by *E. gallinarum* (28–30), strain-specific barrier disruption and T cell dysregulation exemplified by *R. gnavus* (31–33), and metabolite-associated effects linked to *S. intermedius* (34), potentially through acylcarnitine metabolism alterations. This heterogeneity may help explain why no single taxon is consistently enriched across all SLE cohorts, while still supporting the involvement of the gut microbiota in lupus pathogenesis.

### 2.3 Antiphospholipid syndrome

Antiphospholipid syndrome (APS) is a systemic autoimmune disorder characterized by arterial or venous thrombosis and/or pregnancy morbidity with persistent antiphospholipid antibodies. Autoantibodies, represented by β2-glycoprotein I-dependent antiphospholipid antibodies, can promote thrombosis and pregnancy complications through effects on various target cells (35). Because APS is defined by antigen-specific autoantibody responses, microbial molecular mimicry provides a particularly plausible mechanism by which gut microbiota may initiate or amplify autoreactive T- and B-cell responses against β2-glycoprotein I (β2GPI).

A compelling microbiota-associated mechanism in APS is molecular mimicry. Cross-reactivity has been documented between *Roseburia intestinalis* and the T- and B-cell epitopes of the autoantigen β2GPI, which is central to APS pathogenesis (36). This finding establishes a direct mechanistic connection between gut commensal microbes and the autoantibody responses that define the disease. In alignment with this model, oral *R. intestinalis* administration in lupus-prone mice induces anti-β2GPI IgG autoantibodies (36). Further supporting this concept, a recent study identified a YjjG family noncanonical pyrimidine nucleotidase encoded by *Roseburia amylophila* as a candidate β2GPI-mimicking protein, finding increased IgG reactivity against this antigen in patients with APS (37). Although the current evidence is less extensive than that for RA or SLE, the data on APS strongly support the hypothesis that specific commensal microbes can initiate systemic autoreactivity through antigenic mimicry.

### 2.4 Primary sclerosing cholangitis

Primary sclerosing cholangitis (PSC) is a chronic immune-mediated disease characterized by inflammation and fibrosis of the bile ducts, which can lead to progressive liver dysfunction and eventually require liver transplantation. PSC is strongly associated with inflammatory bowel disease, supporting the concept that gut–liver axis dysfunction contributes to its pathogenesis (38). Proposed mechanisms include intestinal barrier disruption and microbial translocation through the portal circulation (39). This close relationship between intestinal inflammation, microbial dysbiosis, and hepatobiliary immune activation provides a rationale for investigating translocating gut pathobionts as disease-modifying factors in PSC.

A recent mucosal microbiome study reported significant enrichment of the genera *Klebsiella*, *Enterococcus*, *Veillonella*, and *Rothia* in colonic mucosal samples from patients with PSC compared with those from healthy controls, consistent with previous findings from stool microbiota studies (40). This mucosa-associated dysbiosis supports the relevance of gut microbial alterations in PSC, although compositional enrichment alone does not establish causality. Among these taxa, *Klebsiella pneumoniae* has been functionally implicated as a pathobiont that links intestinal barrier dysfunction to hepatobiliary inflammation in PSC. Studies utilizing gnotobiotic mice colonized with microbiota derived from patients with PSC have found translocating bacteria in the mesenteric lymph nodes, with *K. pneumoniae* being prevalent (41). This was associated with Th17 cell activation in both the liver and colon, suggesting that gut immune responses can affect distant organs. RORγt inhibition reduced liver injury, highlighting the mechanistic role of the Th17 pathway rather than a mere correlation between microbial imbalance and disease (41).

Caution is warranted when considering the applicability of these findings to other populations. A recent meta-analysis indicated that microbiome associations in rheumatic diseases are not consistently observed and may vary depending on cohort characteristics (42). While the taxa implicated in RA, SLE, APS, and PSC vary, the evidence consistently highlights a limited set of recurring pathogenic mechanisms, including Th17 cell skewing, compromised biological barrier integrity, molecular mimicry, and microbial extraintestinal translocation. This convergence indicates that these clinically distinct autoimmune diseases may share common microbiota-dependent checkpoints, supporting the development of mechanism-based therapeutic interventions.

**Figure 2:**
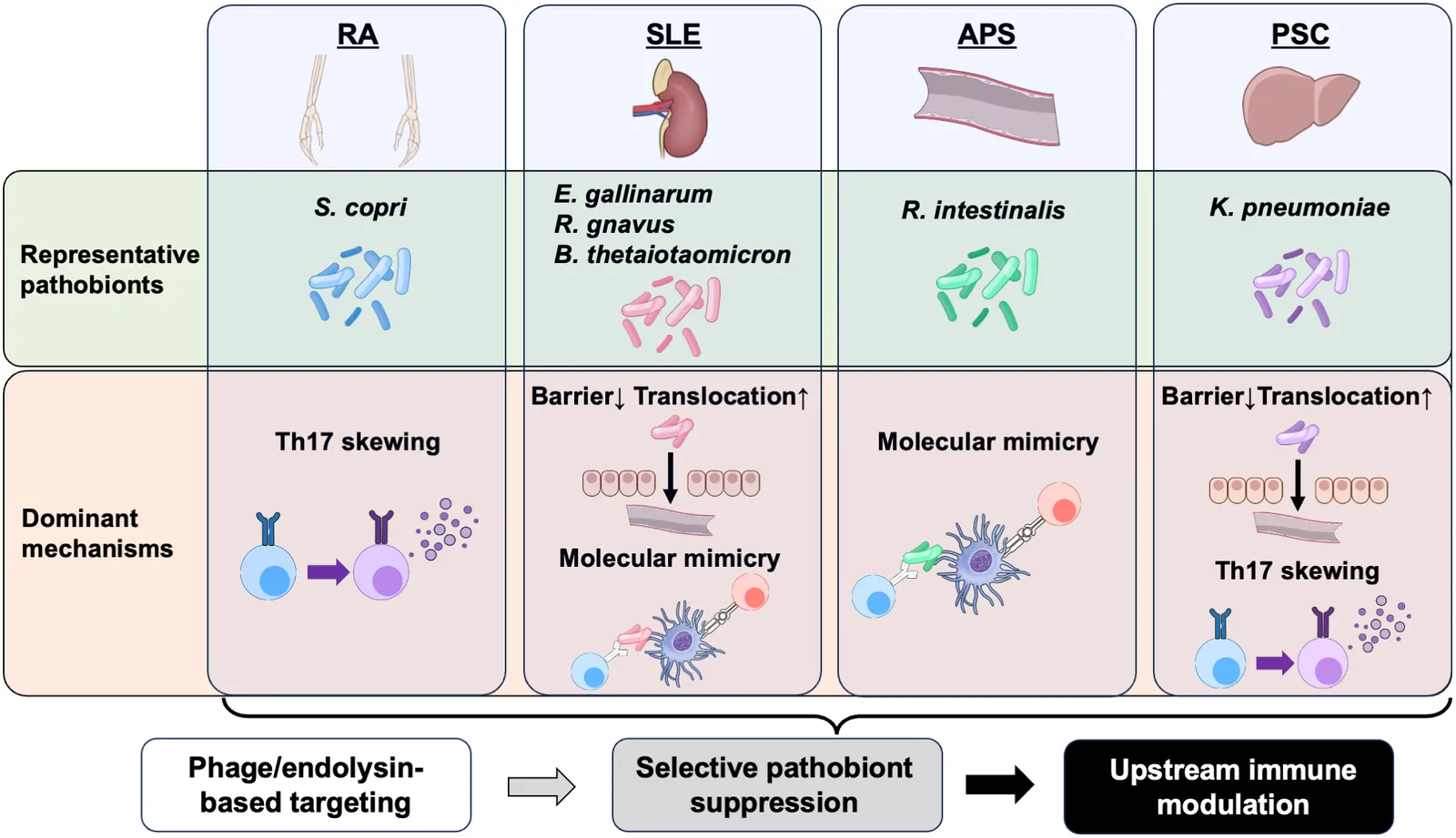
Disease-associated pathobionts, dominant mechanisms, and potential phage and endolysin-based interventions in representative autoimmune diseases. Schematic overview of representative extraintestinal autoimmune diseases discussed in this review. Selected pathobionts include *Segatella copri* in rheumatoid arthritis (RA), *Enterococcus gallinarum*, *Ruminococcus gnavus*, and *Bacteroides thetaiotaomicron* in systemic lupus erythematosus (SLE), *Roseburia intestinalis* in antiphospholipid syndrome (APS), and *Klebsiella pneumoniae* in primary sclerosing cholangitis (PSC); these organisms are linked to partially overlapping mechanisms, including molecular mimicry, mucosal Th17 immune priming, intestinal barrier dysfunction, and bacterial translocation. Pathobiont-directed phage therapy and phage-derived enzymes such as endolysins may enable selective microbial depletion and represent potential precision microbiome-modulating strategies for selected patient subsets. The illustration was created using elements from NIAID NIH BioArt Source entries 41, 46, 55, 98, 114, 173, 208, 229, 230, 250, and 510.

The disease-specific examples provided above illustrate that pathobionts do not function through a single, universal pathway, instead converging on a collection of immunological programs that link gut colonization with systemic immune dysregulation (**Supplementary Table 2**). These representative disease-associated pathobionts, their dominant mechanisms, and potential phage and endolysin-based interventions are summarized in **Figure 2**. Understanding these common mechanisms is vital for creating interventions that specifically target pathogenic microbe-host interactions without broadly compromising immune function.

## 3 Mechanistic insights into gut microbiota-driven systemic autoimmunity

### 3.1 Molecular mimicry

Molecular mimicry represents a prominent mechanism through which commensal microbes may initiate systemic autoimmunity, establishing antigenic continuity between a microbial epitope and a self-antigen. Ro60 is a ring-shaped RNA-binding protein that forms ribonucleoprotein complexes with approximately 100-nucleotide noncoding Y RNAs. It is highly conserved, with orthologs identified across a broad spectrum of taxa, including vertebrates, bacteria, and phages. In experimental models, immunization with human Ro60 induces intermolecular epitope spreading, resulting in autoantibody production against Ro52, La, Smith, and U1 ribonucleoprotein, which are characteristic of SLE. Greiling et al. identified commensal bacteria harboring Ro60 orthologs in fecal samples from patients with SLE, including *Bacteroides thetaiotaomicron*. Ro60-reactive T cell clones exhibit cross-reactivity with *B. thetaiotaomicron*, and colonization of gnotobiotic mice with this bacterium enhances anti-human Ro60 T- and B-cell responses (43). Alongside the β2GPI mimicry observed in APS (36), these findings suggest that molecular mimicry can connect diverse microbial antigens to distinct autoimmune serologies, offering a plausible explanation for how localized gut immune recognition translates into sustained systemic autoreactivity.

### 3.2 Migration of gut-derived T follicular helper cells into the spleen

Another principal mechanism of microbial involvement in systemic autoimmunity involves the physical redistribution of pathogenic lymphocyte subsets that are primed within the gut-associated lymphoid tissues. Oral administration of fecal material containing segmented filamentous bacteria in the K/BxN arthritis model induces disease, and systemic autoimmune characteristics are observed, such as splenomegaly, the expansion of T follicular helper (Tfh) cells, and elevated serum autoantibody titers. Tfh cell differentiation is initiated in the gut before they subsequently migrate to the spleen, as demonstrated in photoconvertible mouse models (44). These gut-derived Tfh cells originate from Th17 cells, indicating T-cell plasticity. Gene expression signatures of atypical Tfh cells have been identified in patients with RA (45). These findings demonstrate that the gut serves not only as a site of microbial sensing but also as a location where pathogenic helper programs are imprinted before being systemically disseminated. Collectively, these findings suggest that systemic autoimmunity may be initiated in gut-associated lymphoid tissues and propagated through pathogenic T cell subset migration.

### 3.3 Apoptosis of regulatory T cells

Pathobionts can facilitate disease progression by interfering with the regulatory systems controlling inflammation. The *R. gnavus* strain RG2 triggers apoptosis in regulatory T cells when cultured with dendritic cells, and its colonization promotes autoantibody production and T cell dysregulation in lupus-prone mice (33). In this context, *R. gnavus* does more than just deliver proinflammatory ligands; it actively undermines a vital tolerogenic compartment, reducing the threshold for ongoing autoimmunity.

### 3.4 Intestinal barrier dysfunction

Intestinal barrier dysfunction, often termed “leaky gut,” is a pivotal host factor that transforms a localized microbial signal into systemic immune activation. Toll-like receptor 7 signaling, a significant pathway in lupus pathogenesis (46), increases intestinal permeability (47), suggesting that host-intrinsic autoimmune susceptibility can directly compromise barrier integrity. *E. gallinarum* increased intestinal permeability in SLE models as measured by FITC–dextran assays, whereas wild-type mice exhibited limited bacterial translocation (28). Similarly, *R. gnavus* derived from patients with SLE possesses a structurally distinct, highly immunogenic lipoglycan that is proposed to enhance gut permeability, facilitating systemic exposure to bacterial products (32). Consistently, lupus-prone mice display increased gut permeability, with severity varying according to genetic background. These findings support a bidirectional model in which host susceptibility and microbial virulence collaborate to destabilize epithelial barriers and amplify systemic autoimmunity.

### 3.5 Bacterial translocation

Bacterial translocation is crucial in the progression from dysbiosis to systemic disease, as it allows direct interaction between extraintestinal tissues, immune cells, and pathobionts or their byproducts. This process has been observed in SLE, where *E. gallinarum* has been found beyond the intestinal barrier (28), and in PSC, where *K. pneumoniae*, *Proteus mirabilis*, and *E. gallinarum* were isolated from mesenteric lymph nodes (41). These findings highlight translocation as a mechanistic checkpoint, rather than a secondary occurrence, linking barrier dysfunction to organ-specific inflammation.

These observations support a comprehensive model in which pathobionts drive systemic autoimmunity through a series of sequential and partially overlapping stages: antigenic mimicry, gut immune priming, regulatory pathway erosion, barrier disruption, and dissemination to extraintestinal sites. These models clarify how taxonomically diverse microbes can lead to similar disease phenotypes and provide a mechanistic foundation for therapies that selectively target and neutralize disease-associated gut microbes. Beyond these mechanisms, the role of gut-associated B-cell responses in the initiation and maintenance of systemic autoimmune diseases remains unknown.

Taken together, these disease-specific mechanistic findings support the concept that pathobionts may serve as therapeutic targets. Because these mechanisms act at upstream checkpoints of autoimmune pathogenesis, selective suppression of disease-associated pathobionts could modify immune pathways before systemic autoimmunity becomes established.

## 4 Phage therapy for chronic immune-mediated diseases

The mechanistic framework described above raises an important therapeutic question: if a limited number of pathobionts act as upstream drivers of chronic immune-mediated diseases, can their selective elimination interrupt disease-promoting host–microbe interactions and thereby mitigate systemic autoimmunity? Phage therapy has emerged as a potential strategy for targeting disease-associated pathobionts and may offer a novel approach for treating chronic immune-mediated diseases that require long-term management. This concept has led to growing interest in precision microbiome therapy, in which disease-associated microorganisms are selectively targeted while the broader microbial ecosystem is preserved. Phage therapy is attractive because it offers the possibility of precise bacterial depletion with a lower risk of dysbiosis compared with antibiotics. This strategy may modulate upstream host–microbe interactions while avoiding the broad systemic immune suppression associated with many current therapies. Rather than broadly suppressing host immunity, pathobiont-directed phages have the potential to intervene upstream of inflammatory cascades by removing microorganisms that initiate or sustain disease-associated immune activation. Although the evidence remains limited, proof-of-concept studies in IBD and PSC suggest that pathobiont-directed phages can ameliorate inflammation *in vivo*. These studies also indicate that therapeutic readiness differs substantially among individual pathobionts, reflecting differences in disease causality, bacterial biology, and the availability of tractable phage platforms. The following sections therefore evaluate current evidence from the perspective of organism-specific translational feasibility.

### 4.1 Preclinical proof-of-concept for pathobiont-directed phage therapy

Among the autoimmune disease–associated pathobionts discussed above, *K. pneumoniae* currently provides the strongest proof of concept for pathobiont-directed therapeutic intervention, as disease association, mechanistic validation, and successful selective depletion have all been demonstrated experimentally. These studies establish a direct translational link between the mechanistic framework outlined in Section 3 and microbiome-targeted therapeutic strategies.

*K. pneumoniae* has been identified as a pathobiont in a subset of patients with IBD. A phage cocktail specifically targeting *K. pneumoniae* has demonstrated efficacy in ameliorating dextran sulfate sodium-induced colitis in gnotobiotic mice colonized with patient-derived *K. pneumoniae*, illustrating that the selective depletion of a disease-associated commensal microbe can influence intestinal inflammation (48). Although IBD is not the primary focus of this review, these findings provide important proof of principle that precise microbiome editing with bacteriophages can modulate immune-mediated disease without broadly disrupting the resident microbiota. This study provides significant proof of principle that microbiome editing through phages is feasible, even within the context of complex mucosal diseases.

The PSC model further substantiates the therapeutic significance of targeting *K. pneumoniae*. A phage cocktail specifically targeting PSC-derived *K. pneumoniae*, administered both orally and intravenously, mitigated disease severity in *Klebsiella*-colonized PSC-model mice (49). Given that PSC pathogenesis in this model is associated with bacterial translocation and Th17 activation, the therapeutic efficacy of phage treatment underscores that the bacterium acts as a disease driver rather than merely serving as a passive biomarker, advancing the field towards causal microbiome intervention. Importantly, because this same pathobiont was previously shown to promote intestinal barrier dysfunction, bacterial translocation, and Th17-mediated hepatobiliary inflammation (41), its successful therapeutic depletion provides functional support for a causal role of *K. pneumoniae* in disease pathogenesis rather than a simple association. Among the autoimmune disease–associated pathobionts discussed in this review, *K. pneumoniae* therefore represents the most advanced example integrating disease association, mechanistic validation, and successful pathobiont-directed intervention.

These preliminary studies indicate that phage therapy may function upstream of inflammation by eliminating disease-promoting taxa rather than broadly suppressing host immunity. However, several challenges must be addressed before its widespread implementation, including host immune responses against phages, limited host ranges, and the potential emergence of resistance. The innate and adaptive immune recognition of administered phages may affect tissue persistence, effective dosing, and the durability of repeated treatments.

Furthermore, the current evidence is derived predominantly from gnotobiotic or otherwise simplified colonization models. Although these systems provide compelling mechanistic insight and proof of concept, they cannot fully recapitulate the ecological complexity, strain-level diversity, and interindividual variability of the human gut microbiome. The effects of selectively depleting candidate pathobionts should be evaluated using appropriate experimental controls and interpreted together with comprehensive analyses of these factors. Such integrated analyses will be essential for distinguishing direct effects of pathobiont depletion from broader ecological changes within the gut microbiome.

### 4.2 Current availability and organism-specific technical challenges

At present, no approved or clinically established phage-based therapy exists for the autoimmune diseases discussed in this review. The available data should therefore be viewed as representing distinct stages of preclinical development rather than a uniform therapeutic landscape. As highlighted in the previous section, therapeutic readiness differs substantially among disease-associated pathobionts, reflecting variation in the strength of causal evidence, bacterial biology, and the availability of suitable phage or phage-derived platforms.

Among the organisms discussed here, *K. pneumoniae* in PSC represents the most advanced therapeutic target. Disease causality is supported by mechanistic studies demonstrating bacterial translocation and Th17-mediated inflammation, and selective depletion using phage cocktails has already shown therapeutic efficacy in preclinical models (41, 49). Consequently, *K. pneumoniae* currently provides the clearest example of successful translation from pathobiont identification to targeted microbiome intervention.

By contrast, translation remains considerably more challenging for the other autoimmune disease–associated pathobionts. *S. copri*, a candidate pathobiont in RA, exhibits substantial strain-level heterogeneity, with only selected strains carrying disease-associated genetic elements such as conjugative transposons. Therapeutic targeting therefore requires strain-resolved characterization and phages capable of discriminating pathogenic from non-pathogenic strains. In addition, its strict anaerobic lifestyle complicates bacterial cultivation, phage isolation, and host-range evaluation.

Similarly, although *E. gallinarum* has compelling mechanistic evidence implicating it in SLE via intestinal translocation and immune activation, no phage-based intervention has yet been reported. As a Gram-positive organism, it may ultimately be amenable to either whole-phage therapy or phage-derived endolysins; however, therapeutic feasibility remains to be established experimentally.

Other candidate pathobionts are at an even earlier stage of therapeutic development. For example, *R. gnavus* shows marked genomic and phenotypic diversity among clinical isolates, making the identification of broadly targetable strains difficult. Likewise, the proposed contribution of *R. intestinalis* to APS is currently supported primarily by molecular mimicry, with limited direct evidence that selective bacterial depletion modifies disease. These organisms therefore remain important mechanistic targets, but additional validation is required before pathobiont-directed therapy can be rationally developed.

Phage targeting generally requires the isolation of lytic phages against disease-relevant strains, because phage susceptibility is often highly strain dependent and cannot be reliably inferred from species-level taxonomy alone (10). This is particularly important for gut commensals, in which pathogenicity may be restricted to specific strains or community contexts. Moreover, several candidate pathobionts discussed in this review are anaerobic, making culture-based isolation, phage screening, and host-range testing technically demanding. Oral delivery further requires that phages or phage-derived enzymes remain active in the intestinal environment and reach the ecological niche occupied by the target organism at sufficient concentrations, as suggested by preclinical and human gut phage studies (48, 49). Gram-negative pathobionts may also be difficult to target with endolysins because their outer membrane restricts access to peptidoglycan, a limitation discussed further below. Finally, targeting a single organism may be insufficient when disease is driven by microbial consortia, microbial metabolites, or host-specific microbiome configurations.

Collectively, these considerations indicate that successful clinical translation will require more than the identification of disease-associated bacteria. Rather, it will depend on rigorous strain-level target validation, the availability of tractable phage or endolysin platforms, individualized or broadly validated phage cocktails, and integration of longitudinal microbiome analyses with functional immune and metabolic readouts. Accordingly, the feasibility of pathobiont-directed phage therapy should be evaluated on an organism-by-organism basis rather than as a universally applicable therapeutic strategy.

### 4.3 Ecological and translational considerations

Potential off-target ecological effects also require careful consideration. Although phages can selectively suppress target bacteria, their ecological consequences may extend beyond direct pathobiont lysis (10, 48, 49). Phage treatment may affect susceptible non-pathogenic strains, reshape microbial interactions, select for resistant bacterial variants, or interact with the resident gut phageome. Future pathobiont-directed phage interventions should incorporate strain-level host-range testing, exclusion of temperate or virulence-associated phages, longitudinal metagenomic and viromic monitoring, and functional assessment of microbial metabolites and host immune responses.

Translating pathobiont-directed phage therapy into autoimmune disease treatment will require a framework distinct from that used for acute bacterial infections. Given the heterogeneity of microbiome associations across autoimmune diseases, patients will need to be stratified according to the presence of target pathobionts and immune features such as autoantibody profiles, gut barrier dysfunction, or Th17/Tfh-associated signatures. Because autoimmune diseases are chronic, repeated administration, durability of bacterial depletion, emergence of resistance, anti-phage or anti-enzyme immune responses, and long-term ecological effects will require careful evaluation (1, 3, 4, 10, 48, 49). Overall, pathobiont-directed phage therapy is best viewed at present as a precision microbiome-modulating strategy for selected patient subsets in whom reducing broad immunosuppression may be desirable.

## 5 Microbiome-targeted therapeutic strategies and phage-derived precision approaches

### 5.1 Microbiome-targeted therapeutic strategies

Although this review focuses on phage-based approaches, pathobiont-directed therapy should be considered within the broader landscape of microbiome-targeted therapeutics, including probiotics, prebiotics, dietary and metabolite-based interventions, fecal microbiota transplantation, defined live biotherapeutic products, and emerging microbiome-engineering technologies. A recent meta-analysis suggested that gut microbiota-based therapies may improve symptoms and inflammatory markers in several autoimmune and rheumatic diseases, although effects were heterogeneous and not consistent in RA or spondyloarthritis (50). Probiotic supplementation has been evaluated in RA, fecal microbiota transplantation has been explored in active SLE, and microbial metabolites such as short-chain fatty acids can regulate epithelial barrier integrity and immune responses (51–53). However, these broader microbiome-restorative approaches generally lack strain-level precision, and fecal microbiota transplantation or donor-derived products require careful donor selection, engraftment assessment, and safety screening as pathogen transmission remains a concern (54). Defined live biotherapeutic products may improve standardization, but require rigorous characterization and manufacturing control (55). Compared with these approaches, phage and endolysin-based strategies may offer higher target specificity, while still requiring careful evaluation of host range, resistance, delivery, immunogenicity, and off-target ecological effects.

### 5.2 Phage-derived enzymes as next-generation pathobiont-targeted therapeutics

Phage therapy presents a promising therapeutic avenue, with growing clinical experience in severe bacterial infections (56); however, its clinical implementation faces several challenges. Host immune responses, including anti-phage antibodies, can reduce therapeutic effectiveness, and the development of resistance in target pathobionts may limit sustained treatment, particularly in chronic autoimmune disorders that may require repeated or prolonged interventions (3, 4, 57). These challenges have spurred interest in phage-derived effector molecules, which retain antibacterial activity while avoiding some of the complexities associated with administering intact phage particles.

Phages are generally categorized as lytic or lysogenic. Traditional phage therapy predominantly employs lytic phages, as lysogenic phages risk DNA integration into host bacterial genomes, potentially facilitating antimicrobial resistance dissemination (10). Advances in metagenomic sequencing have enabled more comprehensive characterization of phage–bacterial interactions and accelerated the discovery of genetically encoded antibacterial tools (6, 58). Among these, endolysins have emerged as promising agents because they can directly degrade bacterial cell walls and lyse target bacteria.

Building on this concept, we developed a pathobiont-targeting endolysin based on metagenomic sequence data and assessed its efficacy both *in vitro* and *in vivo*. This strategy was initially applied to *Clostridioides difficile* infection, where its therapeutic potential was demonstrated (58). We recently extended this approach to acute graft-versus-host disease, a systemic immune-mediated disorder. *E. faecalis* can persist through biofilm formation after allogeneic hematopoietic stem cell transplantation in patients with this condition, complicating its elimination with conventional antibiotics. Using whole-genome sequencing data, we identified an *E. faecalis*-targeting endolysin and demonstrated its bacteriolytic and biofilm-disrupting activity, as well as therapeutic efficacy in gnotobiotic acute graft-versus-host disease model mice colonized with patient-derived *E. faecalis* (59). Endolysin administration did not alter gut microbiota composition, supporting the feasibility of precision depletion of disease-associated bacteria.

These findings suggest that phage-derived enzymes may help bridge mechanistic microbiome research and precision microbial intervention. For autoimmune diseases in which a limited number of pathobionts contribute to disease-relevant immune pathways, such enzymes could provide a complementary strategy to whole-phage therapy by enabling selective bacterial depletion while limiting broader microbiome disruption. Their clinical translation will require careful evaluation of host immune recognition, target specificity, resistance, delivery, and durability of bacterial suppression.

### 5.3 Limitations and unresolved issues of phage-derived enzyme therapy

Although endolysins have shown promising therapeutic efficacy against Gram-positive bacterial pathogens, their activity against Gram-negative bacteria remains limited because the outer membrane restricts access to the peptidoglycan layer (60). This barrier consists of lipopolysaccharides and a lipid-rich bilayer, whose negative charge and hydrophobicity impede enzyme penetration into the bacterial cell wall (61).

Beyond this structural barrier, several additional issues must be considered before endolysin-based approaches can be applied to autoimmune disease-associated pathobionts. As phage-derived protein therapeutics, endolysins can induce anti-endolysin antibody responses (62). Although the clinical implications of these immune responses remain to be fully elucidated, they may influence the pharmacokinetics and therapeutic performance of endolysin-based therapies.

Both bacteriophages and endolysins exhibit target specificity; however, the molecular basis of this specificity differs. While phage host specificity is primarily determined by receptor-binding proteins that recognize specific bacterial surface receptors, endolysin specificity is governed by its catalytic domain and, in many cases, its cell wall-binding domain, which recognize and cleave specific peptidoglycan structures (63, 64). As the antibacterial spectrum of an endolysin does not necessarily mirror the host range of its parent phage, the target spectrum, strain specificity, and potential off-target activity should be evaluated independently for each candidate enzyme.

Although endolysins are generally considered to have a relatively low propensity for resistance because they target highly conserved and functionally essential peptidoglycan structures, reduced susceptibility cannot be excluded, particularly during prolonged or repeated exposure (65–70). While stable, high-level resistance has rarely been observed under experimental conditions, adaptive mechanisms that reduce endolysin susceptibility may emerge and warrant continued investigation. Continuous surveillance and rational engineering strategies will therefore be important during the development of endolysin-based therapeutics.

Proteolytic stability, bioavailability, and target-site delivery remain major translational challenges. Unlike endolysins produced intracellularly during phage replication, exogenously administered endolysins must achieve therapeutically effective concentrations at sites of bacterial colonization. Achieving and maintaining these local concentrations *in vivo* may require relatively high doses and efficient delivery strategies. As protein therapeutics, endolysins may be degraded by host or digestive proteases, cleared rapidly from the circulation, or fail to achieve sufficient concentrations at target sites. Protecting these enzymes from proteolytic degradation while ensuring efficient delivery remains a critical challenge for clinical translation. Strategies such as protein engineering, half-life extension, encapsulation, particle-based delivery systems, live biotherapeutic delivery vehicles, and optimized local administration have been actively explored to improve stability, bioavailability, target-site exposure, and therapeutic efficacy (63, 67, 68, 71–73).

Several engineering approaches have been developed to overcome the outer membrane barrier of Gram-negative pathobionts, including fusion with outer membrane-permeabilizing antimicrobial peptides (such as artilysins) and incorporation of phage-derived receptor-binding domains to facilitate bacterial targeting (60, 74). Further optimization and validation of these strategies will be essential for extending phage-derived enzyme therapy to Gram-negative pathobionts associated with autoimmune diseases.

## 6 Discussion

This review highlights how functionally validated pathobionts may contribute to autoimmune disease progression through molecular mimicry, gut immune priming, intestinal barrier disruption, and microbial translocation, and how these mechanisms may inform phage-based microbiome-targeted interventions. Targeting such disease-associated microbes could provide a strategy to modulate upstream immune pathways with potentially fewer systemic immunosuppressive effects than conventional broad immunosuppression. Phage-based approaches, including phage-derived enzymes, may enable selective suppression of disease-associated taxa while preserving much of the surrounding commensal microbiota. However, this field also raises important immunological questions, including how host recognition of phages or phage-derived agents affects efficacy, persistence, repeat dosing, and the emergence of resistant bacterial variants in non-infectious disease contexts. If successfully translated, pathobiont-directed interventions could shift part of the therapeutic paradigm from generalized immunosuppression toward precision microbiome-based disease modification.

Several important limitations and future directions should be considered. Defining a true pathobiont remains challenging. Disease-associated enrichment of a bacterial taxon alone is insufficient to establish pathogenicity, because microbial effects are highly context-dependent and may vary according to host genetics, immune status, diet, resident microbial communities, and strain-level variation. The term “pathobiont” itself has been used inconsistently, and inappropriate use may obscure the complex host–microbe and microbe–microbe interactions that determine whether a given organism contributes to disease. Future studies should move beyond cross-sectional microbiome-wide associations by incorporating longitudinal sampling, strain-resolved metagenomics, bacterial isolation, gnotobiotic validation, and interventional experiments demonstrating that selective removal or suppression of the candidate organism modifies disease-relevant immune phenotypes to distinguish causal pathobionts from bystander taxa or biomarkers of dysbiosis.

Inter-individual microbiome variability poses a major challenge for translating pathobiont-targeted therapy into autoimmune diseases. Microbiome composition, microbial gene content, and metabolite production differ substantially among individuals and populations, and disease-associated signatures are not always reproducible across cohorts. A systematic review and meta-analysis of rheumatic diseases highlighted that gut microbiome associations are heterogeneous and can be influenced by cohort characteristics and study design (42). More broadly, microbiome-based precision medicine is complicated by person-specific microbial signatures and environmental influences (75). These considerations suggest that pathobiont-directed therapy is unlikely to be universally applicable to all patients with a given autoimmune disease; instead, future clinical translation will require stratification based on the presence of disease-relevant strains, microbial functional activity, immune signatures, and possibly metabolomic or barrier-function readouts. Pathobiont-targeted phage or endolysin therapy may be most appropriately developed as a precision microbiome-modulating strategy for selected patient subsets.

Although animal models, especially germ-free and gnotobiotic mice, are indispensable for testing causality, they have important translational limitations. Mouse models allow controlled manipulation of host genetics, microbial communities, diet, and environmental exposure, providing mechanistic insights that are difficult to obtain in humans. However, murine microbiota, intestinal anatomy, diet, immune development, housing conditions, and colonization dynamics differ from those of humans, and humanized gnotobiotic models do not fully reproduce the ecological complexity of the human gut (76). Recent consensus recommendations also emphasize that preclinical microbiome models are essential but require careful standardization, appropriate bacterial isolates, and improved translational relevance (77). Findings from animal models should be interpreted as a mechanistic proof of concept rather than direct evidence of clinical efficacy. Future studies should combine animal models with longitudinal human cohorts, *in vitro* immune assays, organoid or mucosal culture systems, and early-phase clinical studies to determine whether selective microbial depletion leads to meaningful immunological and clinical improvement in patients.

Finally, regulatory and practical challenges must be addressed before phage-based therapy can be translated into autoimmune diseases. Phage therapy medicinal products require rigorous characterization of phage identity, genome sequence, host range, potency, purity, genetic stability, and absence of undesirable genes, including toxin-, antimicrobial-resistance-, or lysogeny-related genes (78). For autoimmune diseases, additional issues arise because treatment may require repeated or long-term administration. Future development should evaluate anti-phage or anti-enzyme immune responses, durability of target suppression, emergence of resistant bacterial variants, off-target ecological effects on the microbiome and phageome, and standardized clinical endpoints. Regulatory strategies will also need to be adapted to the specific modality, including fixed phage products, individualized phage cocktails, engineered phages, and phage-derived enzymes.

Overall, pathobiont-directed phage-based therapy should not be viewed as a universal replacement for immunosuppression, but as a potential precision microbiome-modulating strategy for selected patient subsets. Its successful translation will require integration of strain-resolved microbiology, mechanistic immunology, safe and scalable product development, and clinically meaningful measures of autoimmune disease activity.

## References

[1] Labrie SJ Samson JE Moineau S . Bacteriophage resistance mechanisms. Nat Rev Microbiol. (2010) 8:317–27. doi: 10.1038/nrmicro2315 20348932

[2] Manrique P Dills M Young MJ . The human gut phage community and its implications for health and disease. Viruses. (2017) 9:141. doi: 10.3390/v9060141 28594392 PMC5490818

[3] Champagne-Jorgensen K Luong T Darby T Roach DR . Immunogenicity of bacteriophages. Trends Microbiol. (2023) 31:1058–71. doi: 10.1016/j.tim.2023.04.008 37198061

[4] Popescu M Van Belleghem JD Khosravi A Bollyky PL . Bacteriophages and the immune system. Annu Rev Virol. (2021) 8:415–35. doi: 10.1146/annurev-virology-091919-074551 34014761

[5] Liu Z Yang Y Mao S Wang Z Zhu Q Yuan Y . Extensive cultivation of human gut phages revealing undescribed Bacteroidaceae phages. Gut Microbes. (2025) 17:2597614. doi: 10.1080/19490976.2025.2597614 41362956 PMC12694917

[6] Nishijima S Nagata N Kiguchi Y Kojima Y Miyoshi-Akiyama T Kimura M . Extensive gut virome variation and its associations with host and environmental factors in a population-level cohort. Nat Commun. (2022) 13:5252. doi: 10.1038/s41467-022-32832-w 36068216 PMC9448778

[7] Pirnay JP Djebara S Steurs G Griselain J Cochez C De Soir S . Personalized bacteriophage therapy outcomes for 100 consecutive cases: a multicentre, multinational, retrospective observational study. Nat Microbiol. (2024) 9:1434–53. doi: 10.1038/s41564-024-01705-x 38834776 PMC11153159

[8] Chan BK Stanley GL Kortright KE Vill AC Modak M Ott IM . Personalized inhaled bacteriophage therapy for treatment of multidrug-resistant Pseudomonas aeruginosa in cystic fibrosis. Nat Med. (2025) 31:1494–501. doi: 10.1038/s41591-025-03678-8 40301561 PMC12092284

[9] Dedrick RM Guerrero-Bustamante CA Garlena RA Russell DA Ford K Harris K . Engineered bacteriophages for treatment of a patient with a disseminated drug-resistant Mycobacterium abscessus. Nat Med. (2019) 25:730–3. doi: 10.1038/s41591-019-0437-z 31068712 PMC6557439

[10] Hatfull GF Dedrick RM Schooley RT . Phage therapy for antibiotic-resistant bacterial infections. Annu Rev Med. (2022) 73:197–211. doi: 10.1146/annurev-med-080219-122208 34428079

[11] Jochum L Stecher B . Label or concept - what is a pathobiont? Trends Microbiol. (2020) 28:789–92. doi: 10.1016/j.tim.2020.04.011 32376073

[12] Lengfelder I Sava IG Hansen JJ Kleigrewe K Herzog J Neuhaus K . Complex bacterial consortia reprogram the colitogenic activity of Enterococcus faecalis in a gnotobiotic mouse model of chronic, immune-mediated colitis. Front Immunol. (2019) 10:1420. doi: 10.3389/fimmu.2019.01420 31281321 PMC6596359

[13] Kullberg MC Ward JM Gorelick PL Caspar P Hieny S Cheever A . Helicobacter hepaticus triggers colitis in specific-pathogen-free interleukin-10 (IL-10)-deficient mice through an IL-12- and gamma interferon-dependent mechanism. Infect Immun. (1998) 66:5157–66. doi: 10.1128/IAI.66.11.5157-5166.1998 9784517 PMC108643

[14] Danne C Ryzhakov G Martínez-López M Ilott NE Franchini F Cuskin F . A large polysaccharide produced by Helicobacter hepaticus induces an anti-inflammatory gene signature in macrophages. Cell Host Microbe. (2017) 22:733–45:e5. doi: 10.1016/j.chom.2017.11.002 29241040 PMC5734933

[15] Surana NK Kasper DL . Moving beyond microbiome-wide associations to causal microbe identification. Nature. (2017) 552:244–7. doi: 10.1038/nature25019 29211710 PMC5730484

[16] Cekanaviciute E Yoo BB Runia TF Debelius JW Singh S Nelson CA . Gut bacteria from multiple sclerosis patients modulate human T cells and exacerbate symptoms in mouse models. Proc Natl Acad Sci USA. (2017) 114:10713–8. doi: 10.1073/pnas.1711235114 28893978 PMC5635915

[17] Girdhar K Huang Q Chow IT Vatanen T Brady C Raisingani A . A gut microbial peptide and molecular mimicry in the pathogenesis of type 1 diabetes. Proc Natl Acad Sci USA. (2022) 119:e2120028119. doi: 10.1073/pnas.2120028119 35878027 PMC9351354

[18] Shen Y Yu X Wang Q Yao X Lu D Zhou D . Association between primary Sjögren's syndrome and gut microbiota disruption: a systematic review and meta-analysis. Clin Rheumatol. (2024) 43:603–19. doi: 10.1007/s10067-023-06754-x 37682372

[19] Gilliland A Chan JJ De Wolfe TJ Yang H Vallance BA . Pathobionts in inflammatory bowel disease: origins, underlying mechanisms, and implications for clinical care. Gastroenterology. (2024) 166:44–58. doi: 10.1053/j.gastro.2023.09.019 37734419

[20] Smolen JS Aletaha D Barton A Burmester GR Emery P Firestein GS . Rheumatoid arthritis. Nat Rev Dis Primers. (2018) 4:18001. doi: 10.1038/nrdp.2018.1 29417936

[21] Holers VM Demoruelle KM Buckner JH James EA Firestein GS Robinson WH . Distinct mucosal endotypes as initiators and drivers of rheumatoid arthritis. Nat Rev Rheumatol. (2024) 20:601–13. doi: 10.1038/s41584-024-01154-0 39251771 PMC12573308

[22] Scher JU Sczesnak A Longman RS Segata N Ubeda C Bielski C . Expansion of intestinal Prevotella copri correlates with enhanced susceptibility to arthritis. Elife. (2013) 2:e01202. doi: 10.7554/eLife.01202 24192039 PMC3816614

[23] Maeda Y Kurakawa T Umemoto E Motooka D Ito Y Gotoh K . Dysbiosis contributes to arthritis development via activation of autoreactive T cells in the intestine. Arthritis Rheumatol. (2016) 68:2646–61. doi: 10.1002/art.39783 27333153

[24] Nii T Maeda Y Motooka D Naito M Matsumoto Y Ogawa T . Genomic repertoires linked with pathogenic potency of arthritogenic Prevotella copri isolated from the gut of patients with rheumatoid arthritis. Ann Rheum Dis. (2023) 82:621–9. doi: 10.1136/ard-2022-222881 36627170 PMC10176341

[25] Maeda Y Rabenow M Xiang W Schmid E Otterbein N Gimaev I . Mucosal innate immune activation as the trigger to Prevotella species-induced arthritis in genetically resistant mice. Cell Rep. (2026) 45:116755. doi: 10.1016/j.celrep.2025.116755 41499240

[26] Tsokos GC . The immunology of systemic lupus erythematosus. Nat Immunol. (2024) 25:1332–43. doi: 10.1038/s41590-024-01898-7 39009839

[27] Silverman GJ Azzouz DF Gisch N Amarnani A . The gut microbiome in systemic lupus erythematosus: lessons from rheumatic fever. Nat Rev Rheumatol. (2024) 20:143–57. doi: 10.1038/s41584-023-01071-8 38321297

[28] Manfredo Vieira S Hiltensperger M Kumar V Zegarra-Ruiz D Dehner C Khan N . Translocation of a gut pathobiont drives autoimmunity in mice and humans. Science. (2018) 359:1156–61. doi: 10.1126/science.aar7201 29590047 PMC5959731

[29] Gronke K Nguyen M Fuhrmann H Santamaria de Souza N Schumacher J Pereira MS . Translocating gut pathobiont Enterococcus gallinarum induces T(H)17 and IgG3 anti-RNA-directed autoimmunity in mouse and human. Sci Transl Med. (2025) 17:eadj6294. doi: 10.1126/scitranslmed.adj6294 39908347

[30] Yang Y Nguyen M Khetrapal V Sonnert ND Martin AL Chen H . Within-host evolution of a gut pathobiont facilitates liver translocation. Nature. (2022) 607:563–70. doi: 10.1038/s41586-022-04949-x 35831502 PMC9308686

[31] Azzouz D Omarbekova A Heguy A Schwudke D Gisch N Rovin BH . Lupus nephritis is linked to disease-activity associated expansions and immunity to a gut commensal. Ann Rheum Dis. (2019) 78:947–56. doi: 10.1136/annrheumdis-2018-214856 30782585 PMC6585303

[32] Silverman GJ Deng J Azzouz DF . Sex-dependent lupus Blautia (Ruminococcus) gnavus strain induction of zonulin-mediated intestinal permeability and autoimmunity. Front Immunol. (2022) 13:897971. doi: 10.3389/fimmu.2022.897971 36032126 PMC9405438

[33] Ma L Ge Y Six N Choi SC Brown J Castellanos Garcia A . Gut expansion of a human lupus pathobiont is associated with autoantibody production and T cell dysregulation. ACR Open Rheumatol. (2025) 7:e70033. doi: 10.1002/acr2.70033 40324961 PMC12052470

[34] Tomofuji Y Maeda Y Oguro-Igashira E Kishikawa T Yamamoto K Sonehara K . Metagenome-wide association study revealed disease-specific landscape of the gut microbiome of systemic lupus erythematosus in Japanese. Ann Rheum Dis. (2021) 80:1575–83. doi: 10.1136/annrheumdis-2021-220687 34426398 PMC8600607

[35] Raschi E Borghi MO Tedesco F Meroni PL . Antiphospholipid syndrome pathogenesis in 2023: an update of new mechanisms or just a reconsideration of the old ones? Rheumatol (Oxford). (2024) 63:SI4–SI13. doi: 10.1093/rheumatology/kead603 38320591

[36] Ruff WE Dehner C Kim WJ Pagovich O Aguiar CL Yu AT . Pathogenic autoreactive T and B cells cross-react with mimotopes expressed by a common human gut commensal to trigger autoimmunity. Cell Host Microbe. (2019) 26:100–13:e8. doi: 10.1016/j.chom.2019.05.003 31227334 PMC8194364

[37] van Mourik DJM Balvers M Jansen VLBI de Jonge PA Coppens M Nieuwdorp M . Cross-reactivity of antiphospholipid antibodies with gut commensal proteins in antiphospholipid syndrome. TH Open. (2026) 10:a28685248. doi: 10.1055/a-2868-5248 42343982 PMC13289573

[38] Manns MP Bergquist A Karlsen TH Levy C Muir AJ Ponsioen C . Primary sclerosing cholangitis. Nat Rev Dis Primers. (2025) 11:17. doi: 10.1038/s41572-025-00600-x 40082445

[39] Hov JR Karlsen TH . The microbiota and the gut-liver axis in primary sclerosing cholangitis. Nat Rev Gastroenterol Hepatol. (2023) 20:135–54. doi: 10.1038/s41575-022-00690-y 36352157

[40] Bajer L Polakovicova P Heczkova M Holm K Hole MJ Hlavaty M . Geography-independent mucosal microbiota alterations in primary sclerosing cholangitis persist after liver transplantation. JHEP Rep. (2026) 8:101716. doi: 10.1016/j.jhepr.2025.101716 41951277 PMC12972987

[41] Nakamoto N Sasaki N Aoki R Miyamoto K Suda W Teratani T . Gut pathobionts underlie intestinal barrier dysfunction and liver T helper 17 cell immune response in primary sclerosing cholangitis. Nat Microbiol. (2019) 4:492–503. doi: 10.1038/s41564-018-0333-1 30643240

[42] Wang Y Wei J Zhang W Doherty M Zhang Y Xie H . Gut dysbiosis in rheumatic diseases: a systematic review and meta-analysis of 92 observational studies. EBioMedicine. (2022) 80:104055. doi: 10.1016/j.ebiom.2022.104055 35594658 PMC9120231

[43] Greiling TM Dehner C Chen X Hughes K Iñiguez AJ Boccitto M . Commensal orthologs of the human autoantigen Ro60 as triggers of autoimmunity in lupus. Sci Transl Med. (2018) 10:eaan2306. doi: 10.1126/scitranslmed.aan2306 29593104 PMC5918293

[44] Teng F Klinger CN Felix KM Bradley CP Wu E Tran NL . Gut microbiota drive autoimmune arthritis by promoting differentiation and migration of Peyer's patch T follicular helper cells. Immunity. (2016) 44:875–88. doi: 10.1016/j.immuni.2016.03.013 27096318 PMC5296410

[45] Fan T Tai C Sleiman KC Cutcliffe MP Kim H Liu Y . Aberrant T follicular helper cells generated by T(H)17 cell plasticity in the gut promote extraintestinal autoimmunity. Nat Immunol. (2025) 26:790–804. doi: 10.1038/s41590-025-02125-7 40307450 PMC12990214

[46] Brown GJ Cañete PF Wang H Medhavy A Bones J Roco JA . TLR7 gain-of-function genetic variation causes human lupus. Nature. (2022) 605:349–56. doi: 10.1038/s41586-022-04642-z 35477763 PMC9095492

[47] Ma L Terrell M Brown J Castellanos Garcia A Elshikha A Morel L . TLR7/TLR8 activation and susceptibility genes synergize to breach gut barrier in a mouse model of lupus. Front Immunol. (2023) 14:1187145. doi: 10.3389/fimmu.2023.1187145 37483626 PMC10358848

[48] Federici S Kredo-Russo S Valdés-Mas R Kviatcovsky D Weinstock E Matiuhin Y . Targeted suppression of human IBD-associated gut microbiota commensals by phage consortia for treatment of intestinal inflammation. Cell. (2022) 185:2879–98:e24. doi: 10.1016/j.cell.2022.07.003 35931020

[49] Ichikawa M Nakamoto N Kredo-Russo S Weinstock E Weiner IN Khabra E . Bacteriophage therapy against pathological Klebsiella pneumoniae ameliorates the course of primary sclerosing cholangitis. Nat Commun. (2023) 14:3261. doi: 10.1038/s41467-023-39029-9 37277351 PMC10241881

[50] Zeng L Yang K He Q Zhu X Long Z Wu Y . Efficacy and safety of gut microbiota-based therapies in autoimmune and rheumatic diseases: a systematic review and meta-analysis of 80 randomized controlled trials. BMC Med. (2024) 22:110. doi: 10.1186/s12916-024-03303-4 38475833 PMC10935932

[51] Alipour B Homayouni-Rad A Vaghef-Mehrabany E Sharif SK Vaghef-Mehrabany L Asghari-Jafarabadi M . Effects of Lactobacillus casei supplementation on disease activity and inflammatory cytokines in rheumatoid arthritis patients: a randomized double-blind clinical trial. Int J Rheum Dis. (2014) 17:519–27. doi: 10.1111/1756-185X.12333 24673738

[52] Huang C Yi P Zhu M Zhou W Zhang B Yi X . Safety and efficacy of fecal microbiota transplantation for treatment of systemic lupus erythematosus: An EXPLORER trial. J Autoimmun. (2022) 130:102844. doi: 10.1016/j.jaut.2022.102844 35690527

[53] Mann ER Lam YK Uhlig HH . Short-chain fatty acids: linking diet, the microbiome and immunity. Nat Rev Immunol. (2024) 24:577–95. doi: 10.1038/s41577-024-01014-8 38565643

[54] DeFilipp Z Bloom PP Torres Soto M Mansour MK Sater MRA Huntley MH . Drug-resistant E. coli bacteremia transmitted by fecal microbiota transplant. N Engl J Med. (2019) 381:2043–50. doi: 10.1056/NEJMoa1910437 31665575

[55] Microbiome Therapeutics Innovation Group Barberio D . Navigating regulatory and analytical challenges in live biotherapeutic product development and manufacturing. Front Microbiomes. (2024) 3:1441290. doi: 10.3389/frmbi.2024.1441290 41853515 PMC12993642

[56] Sawa T Moriyama K Kinoshita M . Current status of bacteriophage therapy for severe bacterial infections. J Intensive Care. (2024) 12:44. doi: 10.1186/s40560-024-00759-7 39482746 PMC11529433

[57] Karamlou S Aghajani E Familsamavati M Azad NF Arayesh S Mohsenipour Z . Bacterial resistance to phage therapy: Mechanisms and strategies to overcome it. Curr Mol Pharmacol. (2025) 18:108–22. doi: 10.1016/j.cmp.2025.11.003 38826717

[58] Fujimoto K Kimura Y Shimohigoshi M Satoh T Sato S Tremmel G . Metagenome data on intestinal phage-bacteria associations aids the development of phage therapy against pathobionts. Cell Host Microbe. (2020) 28:380–9:e9. doi: 10.1016/j.chom.2020.06.005 32652061

[59] Fujimoto K Hayashi T Yamamoto M Sato N Shimohigoshi M Miyaoka D . An enterococcal phage-derived enzyme suppresses graft-versus-host disease. Nature. (2024) 632:174–81. doi: 10.1038/s41586-024-07667-8 38987594 PMC11291292

[60] Wojciechowska M . Endolysins and membrane-active peptides: innovative engineering strategies against gram-negative bacteria. Front Microbiol. (2025) 16:1603380. doi: 10.3389/fmicb.2025.1603380 40529583 PMC12170589

[61] Beveridge TJ . Structures of gram-negative cell walls and their derived membrane vesicles. J Bacteriol. (1999) 181:4725–33. doi: 10.1128/JB.181.16.4725-4733.1999 10438737 PMC93954

[62] Harhala MA Gembara K Nelson DC Miernikiewicz P Dabrowska K . Immunogenicity of endolysin PlyC. Antibiotics (Basel). (2022) 11:966. doi: 10.3390/antibiotics11070966 35884219 PMC9312349

[63] Murray E Draper LA Ross RP Hill C . The advantages and challenges of using endolysins in a clinical setting. Viruses. (2021) 13:680. doi: 10.3390/v13040680 33920965 PMC8071259

[64] Liu H Hu Z Li M Yang Y Lu S Rao X . Therapeutic potential of bacteriophage endolysins for infections caused by Gram-positive bacteria. J Biomed Sci. (2023) 30:29. doi: 10.1186/s12929-023-00919-1 37101261 PMC10131408

[65] Schuch R Nelson D Fischetti VA . A bacteriolytic agent that detects and kills Bacillus anthracis. Nature. (2002) 418:884–9. doi: 10.1038/nature01026 12192412

[66] Fischetti VA . Bacteriophage lysins as effective antibacterials. Curr Opin Microbiol. (2008) 11:393–400. doi: 10.1016/j.mib.2008.09.012 18824123 PMC2597892

[67] Gondil VS Harjai K Chhibber S . Endolysins as emerging alternative therapeutic agents to counter drug-resistant infections. Int J Antimicrob Agents. (2020) 55:105844. doi: 10.1016/j.ijantimicag.2019.11.001 31715257

[68] Love MJ Bhandari D Dobson RCJ Billington C . Potential for bacteriophage endolysins to supplement or replace antibiotics in food production and clinical care. Antibiotics (Basel). (2018) 7:17. doi: 10.3390/antibiotics7010017 29495476 PMC5872128

[69] Abdelrahman F Easwaran M Daramola OI Ragab S Lynch S Oduselu TJ . Phage-encoded endolysins. Antibiotics (Basel). (2021) 10:124. doi: 10.3390/antibiotics10020124 33525684 PMC7912344

[70] Oh J Warner M Ambler JE Schuch R . The lysin exebacase has a low propensity for resistance development in Staphylococcus aureus and suppresses the emergence of resistance to antistaphylococcal antibiotics. Microbiol Spectr. (2023) 11:e0526122. doi: 10.1128/spectrum.05261-22 36862002 PMC10100934

[71] Seijsing J Sobieraj AM Keller N Shen Y Zinkernagel AS Loessner MJ . Improved biodistribution and extended serum half-life of a bacteriophage endolysin by albumin binding domain fusion. Front Microbiol. (2018) 9:2927. doi: 10.3389/fmicb.2018.02927 30538696 PMC6277698

[72] Khan FM Rasheed F Yang Y Liu B Zhang R . Endolysins: a new antimicrobial agent against antimicrobial resistance. Strategies and opportunities in overcoming the challenges of endolysins against Gram-negative bacteria. Front Pharmacol. (2024) 15:1385261. doi: 10.3389/fphar.2024.1385261 38831886 PMC11144922

[73] Pottie I Vázquez Fernández R Van de Wiele T Briers Y . Phage lysins for intestinal microbiome modulation: current challenges and enabling techniques. Gut Microbes. (2024) 16:2387144. doi: 10.1080/19490976.2024.2387144 39106212 PMC11305034

[74] Sisson HM Jackson SA Fagerlund RD Warring SL Fineran PC . Gram-negative endolysins: overcoming the outer membrane obstacle. Curr Opin Microbiol. (2024) 78:102433. doi: 10.1016/j.mib.2024.102433 38350268

[75] Ratiner K Ciocan D Abdeen SK Elinav E . Utilization of the microbiome in personalized medicine. Nat Rev Microbiol. (2024) 22:291–308. doi: 10.1038/s41579-023-00998-9 38110694

[76] Nguyen TL Vieira-Silva S Liston A Raes J . How informative is the mouse for human gut microbiota research? Dis Model Mech. (2015) 8:1–16. doi: 10.1242/dmm.017400 25561744 PMC4283646

[77] Metwaly A Kriaa A Hassani Z Carraturo F Druart C IHMCSA Consortium . A Consensus Statement on establishing causality, therapeutic applications and the use of preclinical models in microbiome research. Nat Rev Gastroenterol Hepatol. (2025) 22:343–56. doi: 10.1038/s41575-025-01041-3 40033063

[78] Fukaya-Shiba A Ogata A Kuribayashi R Sakurai A Suzuki K Takadama S . Regulatory considerations for developing phage therapy medicinal products for the treatment of antimicrobial resistant bacterial infections. Front Pharmacol. (2025) 16:1713471. doi: 10.3389/fphar.2025.1713471 41487519 PMC12756395

