# Supplementary material for: Autoimmune disease-associated pathobionts: mechanisms and therapeutic potential of phage-based approaches
Source: Front Immunol. 2026 Sep 1;17:1884094. doi: 10.3389/fimmu.2026.1884094 (PMC13575821; doi:10.3389/fimmu.2026.1884094)
Supplement: Supplementary file 1 [file Table1.docx]

**Supplementary Material**

**Supplementary Table 1. Representative pathobionts associated with extraintestinal autoimmune diseases.**

Abbreviations: RA, rheumatoid arthritis; SLE, systemic lupus erythematosus; APS, antiphospholipid syndrome; PSC, primary sclerosing cholangitis.

| Disease | Pathobiont | Evidence for pathogenicity | References |
| --- | --- | --- | --- |
| RA | *Segatella copri*  (formerly *Prevotella copri*) | Colonization of SKG mice with *Prevotellaceae*-dominant microbiota exacerbates arthritis | 23 |
| SLE | *Enterococcus gallinarum* | Systemic translocation detected in lupus-prone mice and patients with SLE | 28 |
| SLE | *Ruminococcus gnavus* | Disease-associated expansion and pathogenic effects of patient-derived strains | 31–33 |
| SLE | *Bacteroides thetaiotaomicron* | Ro60 ortholog cross-reactivity and enhanced anti-Ro60 immune responses in gnotobiotic mice | 43 |
| APS | *Roseburia intestinalis* | Oral administration induces anti-β2GPI IgG autoantibodies in lupus-prone mice | 36 |
| PSC | *Klebsiella pneumoniae* | Enrichment and translocation observed in gnotobiotic mice colonized with PSC microbiota | 41 |

**Supplementary Table 2. Mechanisms through which pathobionts contribute to the development of systemic autoimmunity.**

Abbreviations: SLE, systemic lupus erythematosus; APS, antiphospholipid syndrome; RA, rheumatoid arthritis; PSC, primary sclerosing cholangitis.

| Mechanism | Disease/model | Pathobiont | Description | References |
| --- | --- | --- | --- | --- |
| Molecular mimicry | SLE; gnotobiotic mouse model | *Bacteroides thetaiotaomicron* | Ro60 orthologs cross-react with autoreactive T cells, promoting autoimmunity | 43 |
|  | APS | *Roseburia intestinalis* | Mimotopes induce anti-β2GPI IgG autoantibodies | 36 |
| Migration of gut-derived Tfh cells to the spleen | RA,  K/BxN model | *Segmented filamentous bacteria* | Gut-derived Tfh cells migrate to the spleen and enhance autoantibody production | 44, 45 |
| Treg apoptosis | SLE,  B6.NZM model | *Ruminococcus gnavus* | Induces apoptosis of regulatory T cells via dendritic cell interaction | 33 |
| Intestinal barrier dysfunction | SLE | *Ruminococcus gnavus* | R. gnavus-associated increase in gut permeability | 32 |
| Bacterial translocation | SLE, PSC | *Enterococcus gallinarum*, *Klebsiella pneumoniae* | Translocation to systemic sites triggers immune activation | 28, 41 |
